# Supplementary material for: Room-temperature Tamm-plasmon exciton-polaritons with a WSe2 monolayer
Source: Nat Commun. 2016 Oct 31;7:13328. doi: 10.1038/ncomms13328 (PMC5095560; doi:10.1038/ncomms13328)
Supplement: Supplementary Information — Supplementary Figures 1-7, Supplementary Notes 1-6 and Supplementary References [file ncomms13328-s1.pdf]

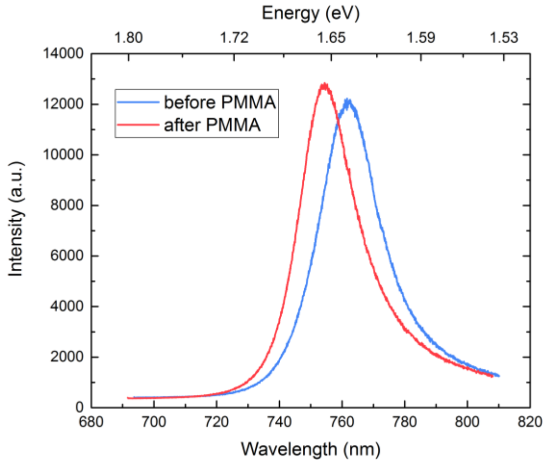

Supplementary Figure 1: Photoluminescence spectra of a WSe_2 monolayer at room temperature before and after capping with PMMA.

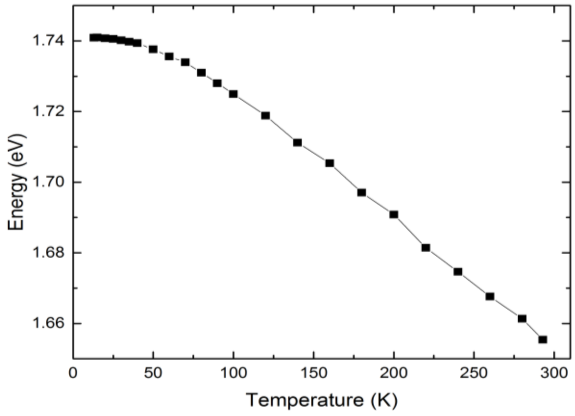

Supplementary Figure 2: Exciton energy as a function of temperature.

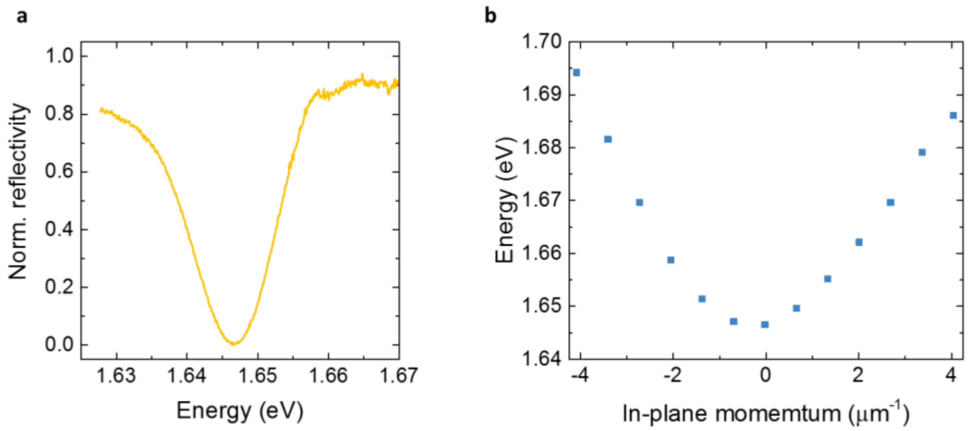

Supplementary Figure 3: a, Normalized reflectivity spectrum of the empty Tamm structure **b,** In-plane dispersion relation of the empty Tamm structure extracted from the angle-resolved reflectivity measurements.

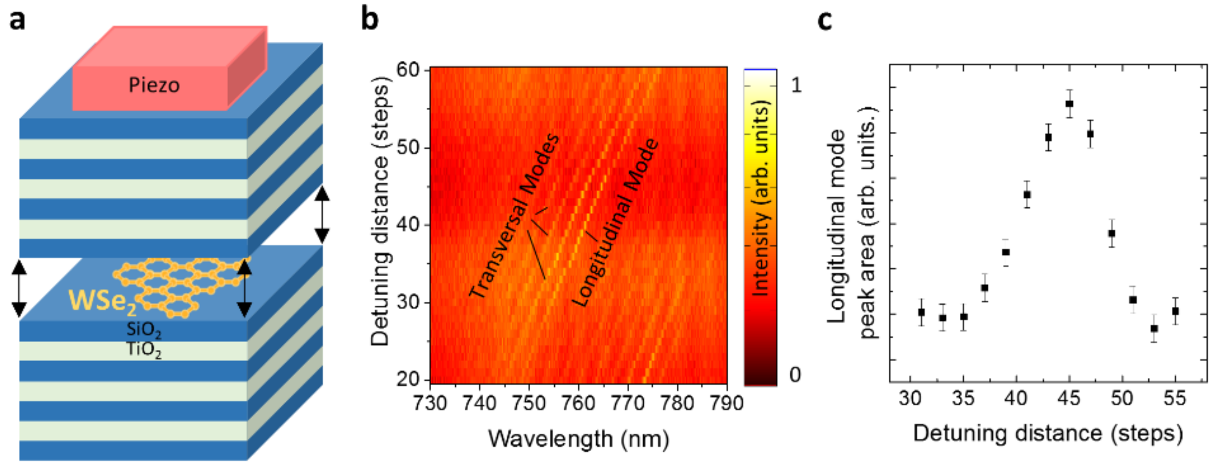

Supplementary Figure 4: a, Schematic illustration of the open cavity design. **b,** Detuning series of the longitudinal and transversal cavity modes. **c,** Integrated PL intensity of the longitudinal mode as a function of the detuning steps. The error bars correspond to standard fitting errors.

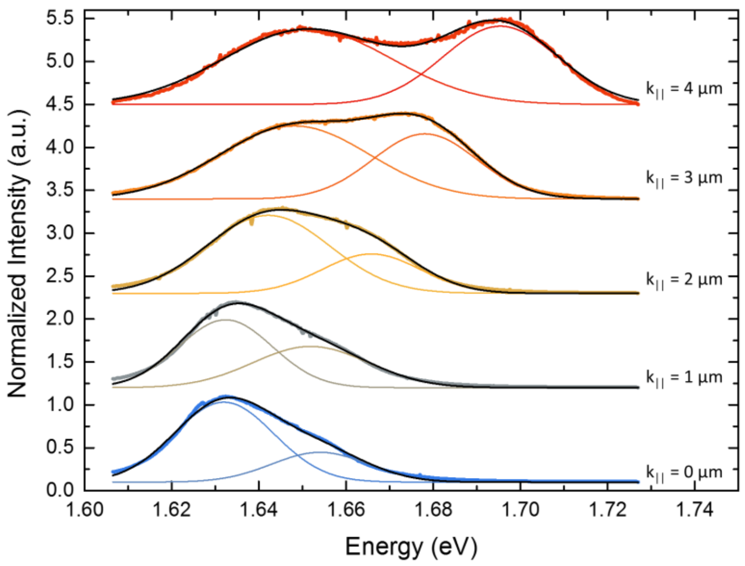

Supplementary Figure 5: Selected photoluminescence spectra taken from Figure 2a including a two Gaussian peak fit and the respective, global fits (black lines).

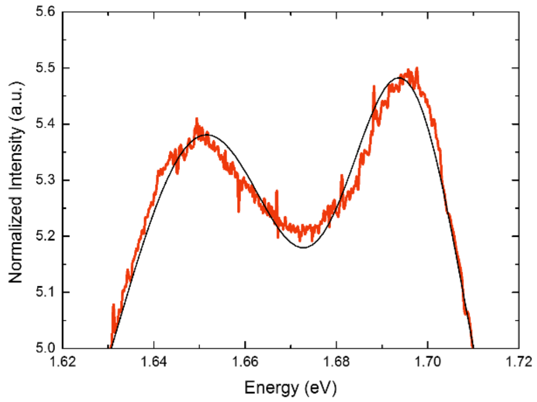

Supplementary Figure 6: Detailed view of a selected photoluminescence spectrum taken from Supplementary Figure 5 ($\mathbf{k}_{\|}$ = 4 µm^{-1}) including the global fit (black line) from a two Gaussian peak fit procedure, which illustrates a slight mismatch between global fit and acquired spectrum.

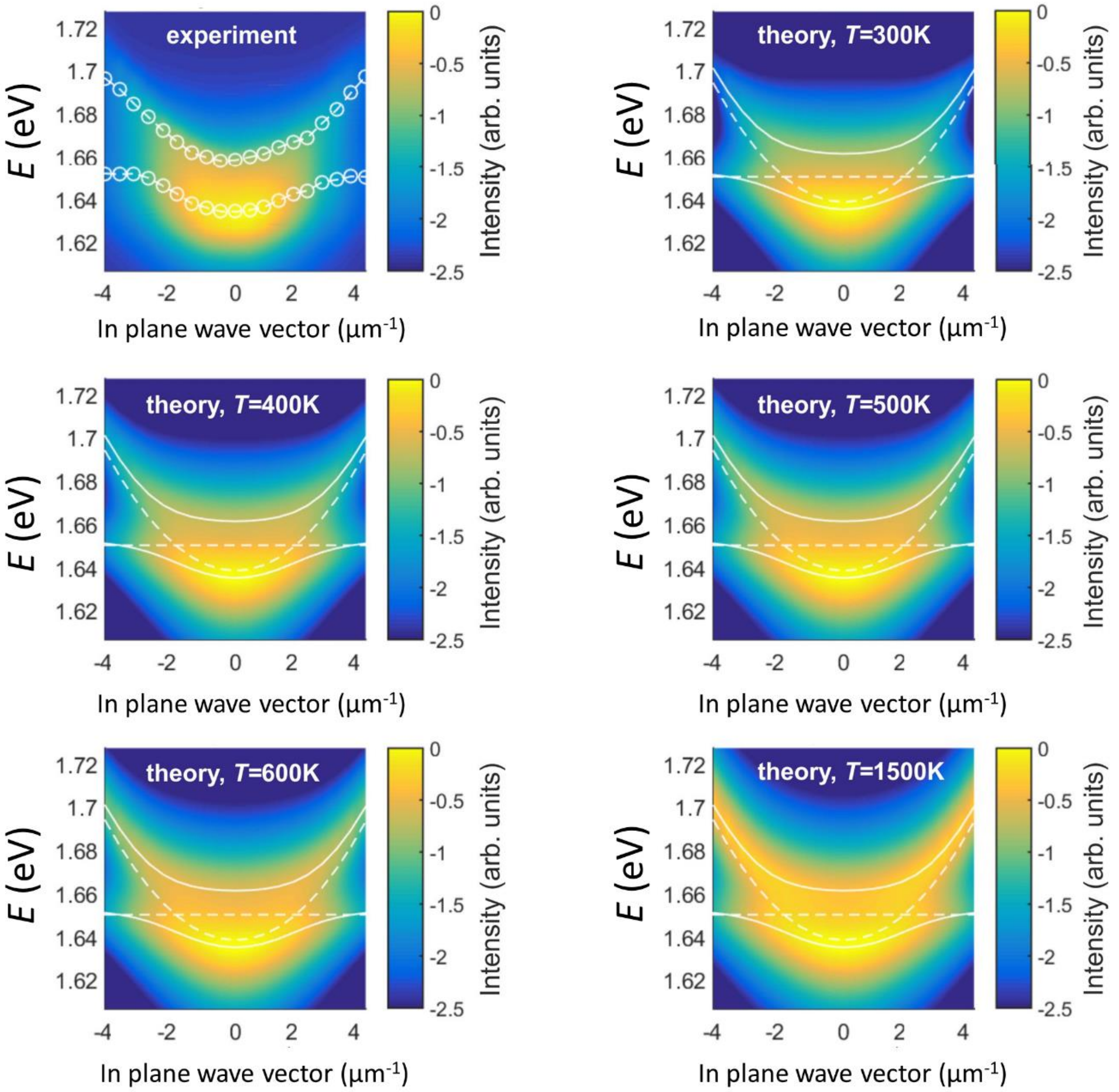

Supplementary Figure 7: Simulated Exciton-polariton momentum-resolved photoluminescence for different temperatures.

Supplementary Note 1: Details on monolayer luminescence

Here, we present the influence of PMMA on the PL emission of WSe_2 monolayers. Supplementary Figure 1 shows the PL spectra of a WSe_2 monolayer that was measured before and after PMMA capping. PL setup adjustments and excitation power (70 µW in front of the microscope objective) were kept the same for both measurements. The peak energy shifts blue by 12 meV and the linewidth decreases from 42.1 meV to 38.7 meV. Therefore, it may well be that the exciton linewidth in the Tamm structure, which is discussed in the mainbody text, is even somewhat smaller than 37.5 meV. The slightly higher linewidth of this flake compared to the one discussed in the main body text is due to the slightly varying optical quality of the individual monolayers. The intensity of the PL is hardly affected by the capping at all.

Additionally, we provide information on the temperature-induced shift of the exciton energy. Supplementary Figure 2 shows the energy-temperature dependence of a separately measured WSe_2 monolayer. Although, the absolute peak energy may vary depending on dielectric environment or strain condition, the measured slope of 0.34 meV/K for high temperatures can be used to calibrate the sample temperature.

Supplementary Note 2: Empty Tamm structure

In this supplementary information section, we present the results of an angle-resolved reflectivity measurement of the Tamm structure without an embedded WSe_2 monolayer. The measurement was taken on the same, completed device close to the monolayer position. Supplementary Figure 2 presents the background corrected and normalized reflectivity spectrum for an in-plane momentum of zero. The linewidth (FWHM) of 15 meV confirms a quality factor of 110. The deviation of 3 meV from the design resonance energy (1.650 eV) can be well explained by slightest variations in the PMMA layer thickness. The dispersion relation of the empty Tamm structure features a parabolic shape in excellent agreement to fitted photonic dispersion in Fig. 2b.

Supplementary Note 3: Control microcavity with a large effective cavity length

We address the case of a control microcavity with a large effective cavity length: Here, we chose an open cavity approach, comprising a nearly identical bottom DBR section as discussed in the main text, and a top DBR attached to piezo actuators to control the physical cavity length, similar to the one discussed in references[1,2]. The device is sketched in Supplementary Figure 3a. We study the PL emission of a WSe_2 monolayer coupled to this cavity resonance by exciting excitons with a non-resonant frequency doubled Nd:YAG laser in CW conditions at a wavelength of 532 nm. The quasi zero-dimensional cavity modes, defined by a macroscopic dimple (curvature radius of 15 µm) in the top DBR are tuned through the exciton resonance by successively varying the cavity length. The effective cavity length is deduced from the longitudinal mode distance and is determined to approximately 8 µm. The distinct crossing behavior between the excitonic and the photonic resonance, as can be inferred from the contour presentation in Supplementary Figure 3b (the excitonic PL emission is hardly visible in this illustration due to the low extraction yield from the high-Q cavity), is a strong evidence of the weak light matter coupling regime. This assumption is verified by plotting the intensity of the fundamental mode from such a cavity with Gaussian confinement as a function of cavity-exciton detuning. We observe a pronounced increase of the luminescence intensity from the cavity mode in the resonance condition, as expected from a device operated in the weak coupling regime (see Supplementary Figure 3c). As pointed out in reference[3], cavities with such high effective cavity length do not provide a sufficient field confinement for room temperature strong coupling of TMDC monolayers.

Supplementary Note 4: Discussion of reflectivity measurements on Tamm-plasmon structure

For more conventional polariton cavities and devices (GaAs, II-VI, organics) reflectivity measurements are an alternative way to map out polariton resonances. However, the Tamm-plasmon structure does not feature a comparably pronounced reflection minimum such as a symmetric Fabry-Perot cavity. This inherently results in weaker reflection signals. Moreover, the size of our monolayer is limited, which makes a very high spatial resolution necessary while preserving a reasonable angular resolution. While this is possible in the photoluminescence configuration, it is demanding in reflection measurements, as increasing spatial resolution always comes to the expense of a strong decrease in the signal to noise ratio. Due to experimental limitations discussed above we have realized a white light reflectivity measurement with a 20x magnification objective with a numerical aperture of 0.40. (Measurements with a higher NA=0.7 objective as for the PL to access higher k-values have not been possible). As in the PL we observe a clear appearance of two normal modes that can be well described by a coupled oscillator model (see main text). The somewhat reduced Rabi splitting measured in reflectivity is expected and in good, quantitative agreement with Savona et al., Solid State Commun. 93, 733 (1997), p. 8-9, eqn. (17)-(21)[4]:

The magnitude of the normal mode splitting in PL reads:

$$\Omega_{\mathrm{PL}} = \sqrt{2\Omega\sqrt{\Omega^2 - 4\Gamma^2} - \Omega^2 - 4\Gamma^2} \qquad \text{Supplementary equation (1)}$$

Whereas in reflectivity, it is written as

$$\Omega_{\mathrm{Refl}} = 2\sqrt{\sqrt{V^4\left(1+\frac{2\gamma}{\gamma_{\mathrm{c}}}\right)^2 + 2V^2\gamma^2\left(1+\frac{\gamma}{\gamma_{\mathrm{C}}}\right)} - \frac{2V^2\gamma}{\gamma_{\mathrm{c}}} - \gamma^2} \qquad \text{Supplementary equation (2)}$$

In these expressions, $\Omega = \sqrt{V^2 - \frac{1}{4}(\gamma_{\mathrm{C}} - \gamma)^2}$ is the Rabi energy related to the coupling constant V and the HWHM of cavity γ_{c} and the exciton γ. $\Gamma = \frac{\gamma+\gamma_{\mathrm{c}}}{2}$ is the average linewidth.

Inserting our experimentally extracted parameters for γ and γ_{c}, for a coupling constant of $V = 27\ meV$ we find a splitting of 23 meV in PL and 15 meV in reflection.

Besides the change in the observed splitting, the excitonic resonance is shifted by 7 meV to lower energies in the reflectivity measurement. This slight shift attributed to sample aging as the reflectivity measurement was taken several weeks after the photoluminescence measurement and the device has gone to several cooling (5K) and reheating cycles during this period. The PMMA film can adsorb water and aceton (used as solvent for the heat conduction silver paste for mounting the sample) vapors and swell under these conditions. A resulting deformation strain of only 0.1% can already explain the energy shift[5].

Supplementary Note 5: Fitting procedure of momentum-resolved spectra

We discuss the detailed fitting procedure of momentum-resolved spectra and the resulting error analysis. Each spectrum was fitted with two Gaussian peaks. Initially, two peak energies are manually selected and then a fitting algorithm (Origin Pro 2015) optimized peak energies, linewidths, intensities and a constant offset. The iterative process minimized the standard deviation of the global fit to a Chi value of below 10^{-12}, allowing up to 500 iterations (all fits converged before reaching this limit). Supplementary Figure 3 illustrates the results of this fitting procedure for five selected spectra that are presented in Figure 2a. For each spectrum the experimental data, the fits for each peak and the global fit are shown. For all spectra the global fit exhibits good agreement with the experimental data.

A closer analysis of the individual spectra shows that the peak positions of the global fit do not always perfectly coincide with the experimental data. For example Supplementary Figure 4 shows the uppermost spectrum of Supplementary Figure 3 in detail. This closer analysis suggests an error of approximately 2 meV for the fit. Furthermore, the starting conditions for the fitting process have a slight influence on the final peak energy result. Therefore, we estimate the error for each data point to be 2.5 meV, added as error bars to Figure 2b and c.

Supplementary Note 6: Simulation of momentum-resolved PL emission vs. temperature

In this section we provide additional simulations showing the momentum-resolved PL intensity as a function of temperature. The exciton-polariton gas in microcavities is usually out of thermal equilibrium with the crystal lattice because of the pumping and dissipation processes. In Supplementary Figure 7 we compare the experimental angular distribution of the polariton emission (a) with theoretical simulations performed assigning to the polariton gas an effective temperature ranging from 300 K (b, lattice temperature) up to 1500 K (f). This comparison shows that the polariton gas is not cooled down to the lattice temperature in our experiments, and it may be characterized by an effective temperature of 300-500 K. We note that the emission from the upper polariton branch is amplified due to the high value of the photonic Hopfield coefficient at this branch at high angular momenta.

Supplementary References

1. Schwarz, S. *et al.* Two-dimensional metal-chalcogenide films in tunable optical microcavities. *ACS Nano* **14,** 7003–7008 (2014).

2. Dufferwiel, S. *et al.* Exciton-polaritons in van der Waals heterostructures embedded in tunable microcavities. *Nat. Commun.* **6,** 8579 (2015).

3. Lundt, N. *et al.* Monolayered MoSe2 : A candidate for room temperature polaritonics. Preprint at http://arXiv.org/abs/1603.05562 (2016).

4. Savona, V., Andreani, L. C., Schwendimann, P. & Quattropani, a. Quantum well excitons in semiconductor microcavities: Unified treatment of weak and strong coupling regimes. *Solid State Commun.* **93,** 733–739 (1995).

5. Schmidt, R. *et al.* Reversible uniaxial strain tuning in atomically thin WSe_2. *2D Mater.* **3,** 021011 (2016).
